# Overview of Some Recent Advances in Improving Water and Energy Efficiencies in Food Processing Factories

**DOI:** 10.3389/fnut.2019.00020

**Published:** 2019-04-02

**Authors:** Nooshin Nikmaram, Kurt A. Rosentrater

**Affiliations:** ^1^Department of Food and Human Nutritional Sciences, University of Manitoba, Winnipeg, MB, Canada; ^2^Department of Agricultural and Biosystems Engineering, Iowa State University, Ames, IA, United States

**Keywords:** food, energy, water, sustainability, efficiencies

## Abstract

Rapid development of food factories in both developed and developing countries, owing to continued growth in the world population, plays a critical role in the food supply chain, including environmental issues such as pollution, emissions, energy and water consumption, and thus food system sustainability. The objective of this study was to briefly review various environmental aspects of food processing operations, including aquatic, atmospheric, and solid waste generation, and also to discuss several strategies that many companies are using to reduce these negative impacts as well as to improve water and energy efficiency. To obtain higher energy efficiencies in food processing factories, two key operations can play critical roles: non-thermal processing (e.g., high pressure processing) and membrane processes. For higher water efficiency, reconditioning treatments resulting in water reuse for other purposes can be conducted through chemical and/or physical treatments. With regards to reducing volumes of processing food waste, two approaches include value-added by-product applications (e.g., animal feed) and/or utilization of food waste for energy production. Finally, we present trends for lowering operational costs in food processing.

## Introduction

Ever-increasing population growth has resulted in higher demand for food, which has led to rapid change and expansion in the number of food factories. It was discussed in the 2009 World Summit on Food Security that by 2050, global food production should rise by at least 70% to feed growing populations (anticipated to be 9 billion people) (1). There are different environmental inputs (e.g., land, water, and energy) and outputs through food systems including raw material/agricultural production, food processing, packaging, distribution, retail, consumption, and end of life. Therefore, all food processing—in addition to food production—results in other problematic outputs such as greenhouse gases, wastewater, as well as packaging and food waste.

Key environmental impacts from food include aquatic, atmospheric, and solid waste generation, which are influenced by the quantity of resources utilized (including energy and water), waste generated, and transport used (truck, train, plane, etc.) in the food system (2). These environmental changes are expected to affect food security and contribute to decreasing the quantity, quality and affordability of food all around the world (3). Management of energy, water, and other resources can lead to increases in efficiency, cost savings, and the minimizing of negative environmental impacts (2).

Energy efficiency policies, which are closely linked to several issues including energy security, climate change, and economic objectives have been paid attention recently (4). For higher energy efficiency achievement, several approaches could be used: firstly, replacement of conventional methods with new technologies (e.g., application of high-pressure processing instead of conventional heating); secondly, membrane processing application instead of energy-intensive operations (e.g., evaporation); and finally, using some technologies for energy production from food waste such as biological, thermal, and thermochemical technologies. The purpose of this review paper is to highlight some environmental impacts of food processing and to discuss some solutions to address these issues.

## Environmental Impacts of Food Processing

### Aquatic Effects

Water is a vital component in the majority of food processing, and its consumption is of great consideration owing to the high-quality water utilized during the manufacture of food products as well as generation of significant volumes of pollutant wastewater. Domestic and industrial water demand have been increasing due to population growth, demand for products, and economic growth, as well as dietary changes into higher animal protein consumption (5). It was determined that a meat-based diet has a larger water footprint (~36% larger) compared to a vegetarian diet (6). For example, the production of 1 g of animal protein from egg, milk, or meat requires ~29, 31, or 112 L of water, respectively; however, 1 g of cereal protein requires 21 L of water (7).

During food processing operations, water is used in many unit operations and applications, including as an ingredient, an initial and intermediate cleaning source, or as an efficient transportation mechanism for some raw materials, and is a key agent utilized in sanitizing plant equipment and areas. Water use will likely continue to be a critical component of the food industry, but it has become a target for efficiency and reduction efforts (8).

Agriculture and food processing can affect water quality via chemistry (e.g., heavy metals, including lead, arsenic, iron, etc.) and bacterial aspects (such as coliforms and *Streptococci* spp.) (9). When discharged into the environment, water containing chemical and/or microbial pollution can negatively impact aquatic life. Heavy metals are a risk to fish, and subsequently to human health (10)—so much so that limits for consumption of some types of fish have been recommended by health agencies. Furthermore, irrigation of crops with polluted wastewater may be problematic, as absorption of the pollutants by growing vegetables, fruits, or other crops, may ultimately lead to contaminants becoming part of the human food supply chain, and thus wastewater may actually be considered a risk factor for human health (9).

### Atmospheric Effects

The main reason for atmospheric emissions from the food industry is extensive energy usage. The majority of energy consumption occurs during the heating of buildings, powering different processes, sterilization, transportation of raw materials and products, and other unit operations. Use of conventional fossil fuels may decrease through increasing the use of renewable energy (e.g., geothermal, wind, or solar energy) (11). Emissions of CO_2_ are predicted to rise significantly in the next 20 years if the production and use of traditional energy via the burning of fossil fuels continues to increase. Increasing levels of CO_2_ in the atmosphere eventually overwhelm the natural carbon cycling by oceans and forests and have driven atmospheric CO_2_ concentrations far above pre-industrial levels. It is thus probable that global temperatures will increase by at least 1.0–3.5°C (12).

Another key parameter leading to atmospheric pollution from the food industry is product transport. The effect of transport depends on various parameters such as the mode of transport, the type, age and condition of vehicles, and the delivery distance.

Further, agricultural activities (which produce most of the raw food products) lead to various air emissions, which will further exacerbate global warming, and include emissions of ammonia, methane, nitrous oxide, as well as sulfur and dust particulate, especially PM_10_ (a mixture of dust, smoke, soot, salt, acids, metals, and other fine particles) (13). Additionally, during fermentation and decomposition of organic materials, as well as during combustion of fossil fuels, volatile organic compounds (VOCs) are produced, which can contribute to ozone formation when combined with nitrogen oxides (NOx) and sunlight (14, 15). Some VOCs also result in negative health effects such as eye, nose, and throat irritation.

Food processing and packaging of raw materials can cause significant air pollution. Apart from air emissions due to fossil fuel combustion, indoor organic dust pollution is unique to this sector (16). Dutkiewicz et al. (17) carried out a study in which air samples for the determination of concentrations of microorganisms, dust and endotoxin were collected at 6 sites in the division producing potato flakes and meal from dried potato pulp and at 2 sites in the division producing potato syrup from imported starch. The concentrations of total airborne microorganisms were within a range of 28.3–93.1 × 10^3^ cfu/m^3^. Mesophilic bacteria were dominant at all sampling sites, forming 73.1–98.8% of the total count. Its airborne concentration increased rapidly after the peeling of potatoes and attained maximal values at cutting and blanching (steaming and sulfuration) of potatoes, and at sacking of potato meal. Several studies also confirmed high levels of particles in food processing industries, such as bacteria, endotoxin, and occupational antigens in places like breweries (18) and sugar-beet processing plants (19).

### Solid Waste Generation

It has been reported that ~1.3 billion tons of food products, such as fresh fruits, vegetables, meats, bakery, and dairy products, are lost along the food supply chain (20). Another estimation indicated that in the United States, about 40% of food produced is lost as waste during processing and distribution by retailers, restaurants, and consumers (21). Additionally, it has been projected that food waste in the European Union will increase from 89 million tons in 2006 to 126 million tons in 2020 (22).

Food processors are substantial waste producers, as they can generate considerable quantities of solid waste (food waste, packaging, etc.) and various liquid effluents (23). These processing wastes can include fruit and vegetable residues, discarded fruits and vegetables, molasses and bagasse from sugar refining, bones and blood from meat and fish processing, non-fermentable residues from wineries, distilleries, and breweries, wastes from dairy factories (e.g., cheese whey), and wastewaters from various unit operations such as washing, blanching, and cooling (24). The disposal of these types of food wastes into the environment should be avoided due to several reasons, including poor biological stability, considerable concentrations of organic components, poor oxidative stability, and significant nutritional value which can be lost from the human food chain. Additionally, a high amount of food waste coupled with microbial decomposition can result in adverse effects on the environment as well as human health (25). To minimize environmental burdens due to food waste, and also to lower the risks to human health, proper management, recycling, and value-added applications are necessary (24).

Many food wastes mostly consist of various carbohydrates (such as starch, cellulose, and hemicellulose), lignin, proteins, and lipids, as well as various organic acids and minerals (ash). Due to the generally high carbohydrate composition of these wastes, the production of renewable energy may be a viable alternative to landfilling. Apart from energy generation, most food wastes contain compounds that could be used as substrates and nutrients for various microbial and enzymatic processes (26, 27). Additionally, utilization of food waste can lead to improvement in the bottom line for both the company and for the locality, and can lead to lower environmental pollution and/or pressure.

## Strategies for Environmental Impact Reduction

**Figure 1:**
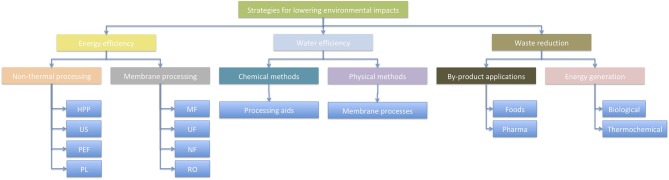
Examples of three options for improving energy and water sustainability in food factories.

There are many opportunities to improve the environmental footprint of food processing operations. Three of these include improving energy efficiency, water efficiency, and waste reduction (Figure 1), which will be discussed in this paper. There are many more that are not considered in this paper, however, and the reader is referred to other papers in this Research Topic [e.g., (28, 29)].

### Energy Efficiency

#### Non-thermal Processing

One challenging topic for all food processing sectors has always been energy efficiency enhancement; illustrating this point, the food industry was classified as the fifth biggest consumer of energy among 20 manufacturing sectors in US in 2002 (30). Depending upon the type of products produced, a great quantity of energy is often applied during the conversion of raw substances into higher-value food products (31). For example, to evaporate 1 kg of water from products, an average of 6 MJ of heat is needed during drying process; however, to reduce the temperature of products under −20°C, 1 MJ (or 0.3 kWh) of electricity is required during freezing processes (8). In this regard, heating processes are often the most energy-intensive types of unit operations used in the food industry, and can include pasteurization, sterilization, dehydration, evaporation, and drying. In conventional heating methods, heat is transferred to the food material via conduction, convection, and radiation heat transfer. Often, the movement of heat from the surfaces of the materials toward their centers is considered a limitation for thermal treatment due to relatively slow heat transfer through food products. However, other effective techniques for heat transfer by using newer technologies (e.g., molecular interactions via microwave) are gaining acceptance (31). Applications of these newer methods not only results in better energy utilization and heat recovery, but can also improve the overall sustainability of food production as well as the nutritional quality of final products (32).

Non-thermal methods are also gaining popularity. These techniques offer several advantages to food manufacturers, such as minimizing the impact on nutritional and sensory properties of food products, they can extend shelf life by preventing or destroying microorganisms, and they can be more energy efficient as well (33). In addition to saving energy by applying these technologies, most of these new approaches also result in water savings, increased reliability, lower energy required (and thus lower emissions), and improved product quality (34). Some of these emerging methods include high pressure processing (HPP), ultrasound (US), pulsed electric fields (PEF), and pulsed light treatment (PL) (35). Table 1 summarizes the effects of the application of several non-thermal technologies on energy efficiency improvement, and will be discussed below.

**Table 1:** Examples of energy efficiency improvements by non-thermal processing applications^*^.

**Method**	**Process conditions**	**Key results**	**References**
HPP	Pressure: 600 MPa Temperature: 20°C Time: 60 s	Compared to thermal pasteurization (65°C for 1 min and 85°C for 25 s), HPP resulted in longer shelf life and lower microbial population over 12 weeks.	(36)
	Pressure: 400 and 600 MPa Temperature: 20°C Time: 5 min	Final apple product of HPP indicated better results including higher fresh-like, value-added products with reasonable shelf life rather than conventional pasteurization (75°C/10 min).	(37)
US	Frequency: 20 kHz Power capacities: 100 w Max time duration: 40 min	US application directly coupled to the food samples led to optimum energy transfer for food dehydration.	(38)
PEF	Temperature: 35–70°C Electric field strength: 8–40 kV/cm Energy input: 5–120 kJ/kg Pulse repetition rate: 2–95 Hz	A significant reduction was observed in energy consumption from 160 to 100 kJ/kg by higher temperature (from 40 to 50°C) during achievement a 7-log10 inactivation of *E. coli*.	(39)
	Temperature: 4–20°C Electric field strength: 30–80 kV cm Pulse frequency: 1–815 Hz Energy input: 0–300 kJ/kg Pulse width: 0.05, 0.1, 0.25, 0.5, 1, 2 and 3 μs	Lower energy consumption (from 44 to 32 kJ/kg) was observed for destruction of *S. enteritidis* through increasing pulse width from 0.05 to 1 μs.	(40)
	Electric field strength: 3–5 kV/cm pulse duration: 1.6 μs 40–80 pulses	Owing to lower force required for a beet slicing by PEF application, total process energy requirement reduced.	(41)
	Electric field strength: pulse duration: 1.5 μs 40–80 pulses	For drying plants such as grass, 50 % energy saving was achieved by PEF rather than traditional methods.	(42)
	Electric field strength: 1.0–2.5 kV/cm Pulse frequency: 100 Hz Pulse width: 30 μs	PEF as a pretreatment led to time reduction of drying the red pepper by ~34.7%.	(43)
		PEF application as a pretreatment for drying crystal radish indicated higher drying rate and lower drying time and energy consumption.	(44)
PL		PL treatment for 3 s resulted in 7.29-log CFU/ml reduction of *E. coli* inoculated in apple juice.	(45)
		The population of *L. innocua* was reduced by 1.39 log CFU using PL treatment and there was no significant growth after 8 days of storage at 4°C.	(46)

* *HPP, high pressure processing; US, ultrasound; PEF, pulsed electric field; PL, pulsed light treatment*.

During HPP, momentary pressure within the range of 300–700 MPa is transmitted throughout the food products, resulting in a reduction of processing time and consequently energy consumption (47, 48). To compare application time and temperature between HPP and conventional processing, (36) reported that HPP (600 MPa/20°C/60 s) resulted in a lower microbial population throughout 12 weeks, and hence a greater shelf life than thermal pasteurization (65°C for 1 min and 85°C for 25 s). Another study found that HPP (400 and 600 MPa/5 min/20°C) was a better alternative for apple processing vs. conventional pasteurization (75°C/10 min) (37). In terms of processes such as chilling and freezing, there is an obvious reason for lower energy consumption during HPP than conventional methods because, during the phase change during HHP, the latent heat of water is nearly 30% lower compared to that at atmospheric pressure (49). Due to water expansion during freezing, pressure increments can lead to lower freezing points (50). HPP can be used to use pressure to induce freezing and thawing, so that the growth kinetics of ice crystals results in a finer crystal structure within the food matrix (51, 52).

**Figure 2:**
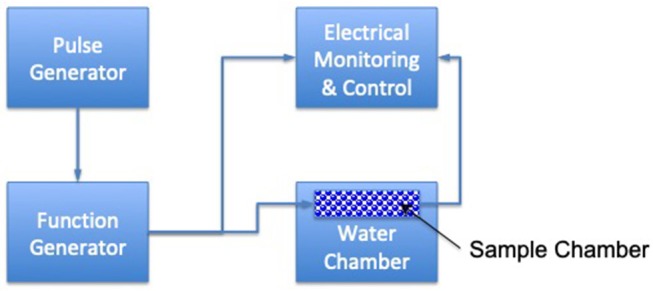
Schematic diagram of ultrasonic velocity continuous wave technique [based upon (56)].

Ultrasound (US) is energy generated by sound waves (53) and has shown high potential for increased heat transfer and faster cooking rates compared to conventional cooking methods (54). The main mechanism of action is cavitation. When air bubbles implode, high localized pressures and temperatures occur, and can reach 50 MPa and up to 5,000°C (55) (Figure 2). Efficacy depends upon the food matrix and upon the intensity of the US; heat transfer improvement of about 30–60% has been seen (57). The main advantage to using US is the fact that temperatures are generally between 40 and 50°C during ultrasonic pasteurization, which are considerably lower than the temperatures used in conventional pasteurization processes. In this regard, it has been reported that *Escherichia coli* and *Saccharomyces cerevisiae* were reduced by more than 99% after ultrasonication, whereas *Lactobacillus acidophilus* was reduced by 72 and 84% depending on the media used (58). Hence, the resistance to ultrasound treatment of spores and Gram-positive and coccal cells is higher than vegetative, Gram-negative and rod-shaped bacteria. Ultrasonication combined with heat was applied to examine the inactivation of *Listeria innocua* and mesophilic bacteria in raw whole milk (59). A combination of US and heat led to an increase in the kill rates compared to the rates of thermal treatment alone, and a synergistic rather than an additive effect was observed. Zhu et al. (60) demonstrated that the use of ultrasound (21.2 kHz, 2 min) enhanced the efficacy of selected sanitizers (such as water, chlorine, acidified sodium chlorite, peroxyacetic acid, and acidic electrolyzed water) in reducing *E. coli* O157:H7 populations in spinach.

Use of US to preserve the nutritional and sensory properties of various food products has been widely evaluated (61–63). In terms of drying processes, combinations of US with moderate heat can result in significant reductions of both processing temperature and processing time compared to the use of air-drying alone. (38) found that the time required to dry carrot slices decreased from 35 min to 25 min when using air-drying alone at 60°C vs. air-drying combined with US at the same temperature. Ortuño et al. (64) conducted an experimental study on the convective drying kinetics of orange peel slabs (thickness 5.95 ± 0.41 mm) at 40°C and 1 m/s with and without power ultrasound application. Obtained data indicated that ultrasonic application influenced both internal and external mass transport. Kek et al. (65) evaluated ultrasound pre-osmotic treatment prior to hot-air drying of guava slices. According to the results, ultrasonic pretreatment lowered the drying time by 17–33%, increased the effective diffusivity by 18–35%, and increased the drying rate constants of guava slices by 37–42%.

In fact, there are several advantages to using US for food processing instead of conventional processing; these may include more effective bulk mixing and micro-mixing, faster heat transfer, better mass transfer, decreased thermal and concentration gradients, reduced processing temperatures, smaller equipment size, faster start-up, smaller production increments, and reduction in the number of processing steps (7).

**Figure 3:**
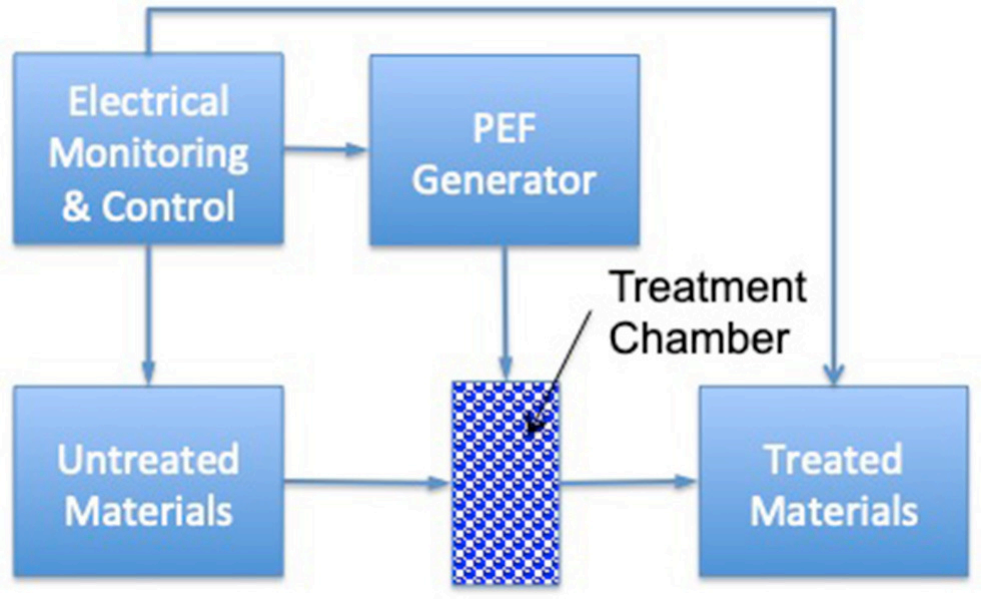
Schematics of a PEF processing system for pumpable products [based upon (66)].

Pulsed electric field (PEF) can be an effective inactivation method for microbial cells when it is combined with low to moderate processing temperatures (<50°C) through inducing permeabilization of biological cells. During this process, tissues are exposed to an electrical field [typically a very short timeframe (μs)] and high-voltage (kV) pulses (Figure 3). Effectiveness depends on the electric field strength, applied temperature, processing time, and energy input (67). Heinz et al. (39) evaluated the effect of temperature (35–70°C) on the lethality of PEF for *E. coli* contamination in apple juice. They observed a negative correlation between energy requirements and treatment temperatures. To obtain a 7-log_10_ inactivation of *E. coli* at 24 kV/cm, the energy requirements declined from 160 to 100 kJ/kg, with a temperature increment of 40 to 50°C. Additionally, it was reported by Korolczuk et al. (40) that, for *S. enteritidis*, by increasing pulse width from 0.05 to 1 μs during PEF processing (50 kV/cm and 15°C), a lower amount of energy (from 44 to 32 kJ/kg) was required. Further, the application of PEF (3–5 kV/cm, 1.6 μs pulse duration, 40–80 pulses) for sugar beet dehydration led to a lower level of force being required for beet slicing (from 16 to 8 N), which then decreased the total energy requirement for processing (41). Another study also confirmed that PEF (7 kV/cm, 1.5 μs pulse duration, 40–80 pulses) resulted in more than 50 % energy savings compared to traditional methods for the drying of plants (i.e., grass, maize, and lucerne) (42).

PEF has been used before and during drying processes because of improved mass transfer and localized structural changes of cell membranes, as well as an increase in membrane permeability (68, 69). It has been shown that drying of red pepper at 45°C takes 4.9 h, but after PEF pre-treatment (2.5 kV/cm, 100 Hz, 4 s), drying time was reduced by 35% (43). Another recent investigation optimized PEF pre-treatment of radish (1.446 V/cm for 28 μs, and 87 pulse), and found an improvement in the drying rate by 26% and a reduction of the drying time by more than 14%, leading to reduced energy consumption (44). When used in conjunction with conventional drying, PEF pre-treatment resulted in decreased drying time by up to 50%, and drying temperature did not exceed 60°C. A reduction of drying time and/or drying temperature can result in a considerable reduction in energy consumption (70, 71).

Pulsed light (PL) processing is an energy-saving, waste-free and environmentally friendly technology. Light pulses are based on electromagnetic energy, which is accumulated in a capacitor and then released in the form of light within a very short time (ns or ms); therefore, this process results in an amplification of power with a minimum of energy consumption. Several studies have been conducted to determine microbial population reduction for several organisms using PL treatments. For example, *E. coli*-inoculated apple juice was lowered by 7.29-log CFU/ml after 3 s of a PL treatment using 88,000 mJ/cm^2^ (45). Cold pasteurization of milk was treated by PL with a minimum dose of 12.6 J/cm^2^ delivered in 56 s (72). In 2009, Uesugi and Moraru used PL (9.4 J/cm^2^) to reduce *L. innocua* on the surface of sausages, and found a 1.39 log CFU reduction after PL treatment, and found no growth after 8 days of storage at 4°C.

#### Membrane Processes

**Figure 4:**
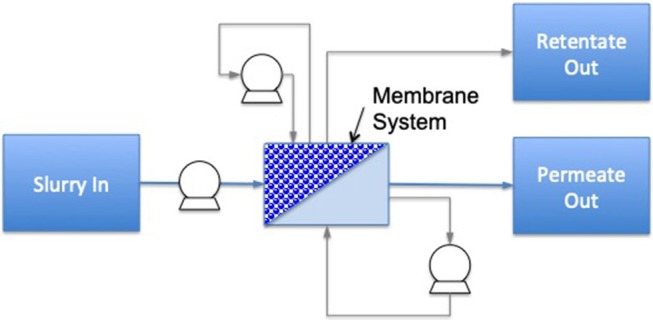
Diagram of a membrane filtration system including cross-flow and flux enhancement. Dotted lines identify the boundaries for system analysis; circles with symbols identify the processing control measurements needed for energy calculations (light gray are optional measurements), the dark gray area illustrates the treatment chamber and darker shapes the food material. Additional system components are named individually [based on (74)].

A highly energy intensive unit operation is evaporation; it is commonly carried out by mechanical vapor recompression technology. However, a good alternative for energy efficiency enhancement is membrane filtration, with a potential energy savings of 30–50% compared to distillation and evaporation (73). The principle of membrane filtration is based on forcing liquid food through a membrane whereby, after a determined processing time, two different streams are obtained, including permeate (material passing through the membrane) and retentate (concentrate rejected by the membrane) (8) (Figure 4). There are four groups of membrane processes based on membrane pore size, consisting of microfiltration (MF) (0.2 to 1 μm), ultrafiltration (UF) (0.02 to 0.1 μm), nanofiltration (NF) (0.001 to 0.01 μm), and reverse osmosis (RO) [<0.001 μm (75, 76)].

Energy consumption of membrane filtration is ~14–36 kJ/kg of water removed, compared to evaporation with mechanical vapor-recompression of 50 kJ/kg of removed water. Processing hot feed and recovering heat in the hot permeate (via heat exchangers) is another energy saving method that is commonly employed with membrane filtration (73). However, one limitation of this process is reported to be relatively low dry weight yields (12–20%), hence a hybrid process consisting of membrane filtration and evaporation is often utilized specially in the dairy processing industry (77). Another potential disadvantage is fouling of the membrane as a result of different compounds, including salts, sugars, proteins, and fats present in the food material. Fouling leads to higher energy consumption and reduced processing efficiency; therefore, to address this challenge, regular cleaning with caustic solutions is typically required (78).

Membrane technology can be applied alone or in combination with other unit operations, such as distillation and evaporation, in order to concentrate various dilute solutions (e.g., grain milling, vegetable oil extraction, sugar manufacturing, etc.). By using membrane systems to remove water in corn wet milling, about 90% energy savings have been reported by Rausch (79) because of no need to provide heat for phase change. Considerable energy (electricity) is needed for pumping to produce high transmembrane pressure and recirculation; thus the total energy balance should be carefully studied (8). However, it has been recently reported that there are new operating conditions that use renewable energy sources coupled with forward/reverse osmosis to promote water recovery from low-strength wastewater. In this regard, anaerobic acidification and forward osmosis (FO) membrane were simultaneously integrated into an air-cathode MFC (AAFO-MFC) for enhancing bio-electricity and water recovery from low-strength wastewater (80). During a long-term operation of ~40 days, the AAFO-MFC system achieved continuous and relatively stable power generation, and the maximum power density reached 4.38 W/m^3^. The higher bio-electricity production in the AAFO-MFC system was mainly due to the accumulation of ethanol resulting from the anaerobic acidification process and the rejection of FO membrane. In addition, a proper salinity environment in the system controlled by the addition of MF membrane enhanced electricity production. These results substantially improve the prospects for simultaneous wastewater treatment and energy recovery. The use of most renewable sources of energy (e.g., hybrid renewable energy sources and battery storage) can also be coupled to produce high transmembrane pressure and recirculation in membrane systems (81). There are other benefits in membrane application, such as selectivity, ease of system operation, lower operating, maintenance, and manufacturing costs compared to heating processes and a better-quality product due to low processing temperatures (i.e., room temperature) (82, 83).

During soybean oil extraction with hexane, the raw extract usually consists of both soybean oil (25–30%) and hexane (70–75%) (84). In a common industrial practice, distillation of the remaining hexane consumes most of the energy cost; however, the application of ultrafiltration or reverse osmosis can lead to substantially reduced energy consumption for hexane evaporation and reduced thermal damage as well (85).

In sugar factories, sugar thin juice obtained from filtration is entered into an evaporation step to concentrate sugar solution. To reduce thermal energy consumption of sugar dehydration, Madaeni and Zereshki (86) used a two-stage RO system for preconcentration of sugar thin juice. They concluded that use of this system prior to final concentration in evaporators resulted in a 33% energy savings.

### Water Efficiency

Pressure on limited water reserves has led to the efforts of many governments and water authorities to improve water use efficiency and to encourage water conservation. There are considerable differences in water consumption amongst various food factories. For example, high water users in the food industry include meat, dairy, and fruit and vegetable processors. By contrast, bakeries and grain producers, which are mostly involved in dry processes, can be categorized as relatively small water users (87). Apart from food processors and their water consumption, there are always some benefits to higher water efficiency.

For example, the Australian Government carried out a survey on manufacturing groups and reported a large savings on total water usage of up to 25, 30, and 60% through the use of basic initiatives such as behavioral changes, water recycling (without conditioning treatment), and water use monitoring, respectively. Other substantial savings were also observed by technology changes (36%) and product redesign (72%), both of which require time and investment (88). The first action before conducting reconditioning treatments for wastewater should be water use reduction, because this approach requires lower training and investment, and the volume and strength of the wastewater are also directly altered (5).

Reconditioning treatments for wastewater include various physical processes, chemical processes, or a combination of both types of treatments. These are commonly used to decrease microbial levels and to eliminate hazardous chemicals in water streams. During chemical treatments, several processing additives may be applied, including chlorine, chlorine dioxide, chloramines, ozone, hydrogen peroxide, or peracetic acid (89). On the other hand, for physical treatments, membrane systems can be used to recover various valuable by-products from wastewater streams (e.g., protein or lactose from whey) (5). Membrane systems for reconditioning wastewater can include microfiltration (MF), ultrafiltration (UF), nanofiltration (NF), and reverse osmosis (RO). These are used for specific ranges of particle sizes. In practice, application of MF for wastewater can separate microbes; UF can be used to remove microbes and suspended solids, however RO can separate microbes, suspended solids, and even some dissolved solids (90).

Water reuse offers great opportunities for lessening groundwater depletion but it does have challenges, such as balancing supply with demand, the risk of potential contamination of stored water with pathogens from wildlife, potential negative effects on crop yields due to higher salinity, potential health concerns associated with contaminants, and public perception of use on food crops (91). Therefore, other actions should also be taken into consideration, such as monitoring pathogens and chemicals, optimization of treatments, assessment of treatment performance, reliability of treatments, etc.

### Waste Reduction

#### By-product Applications

**Figure 5:**
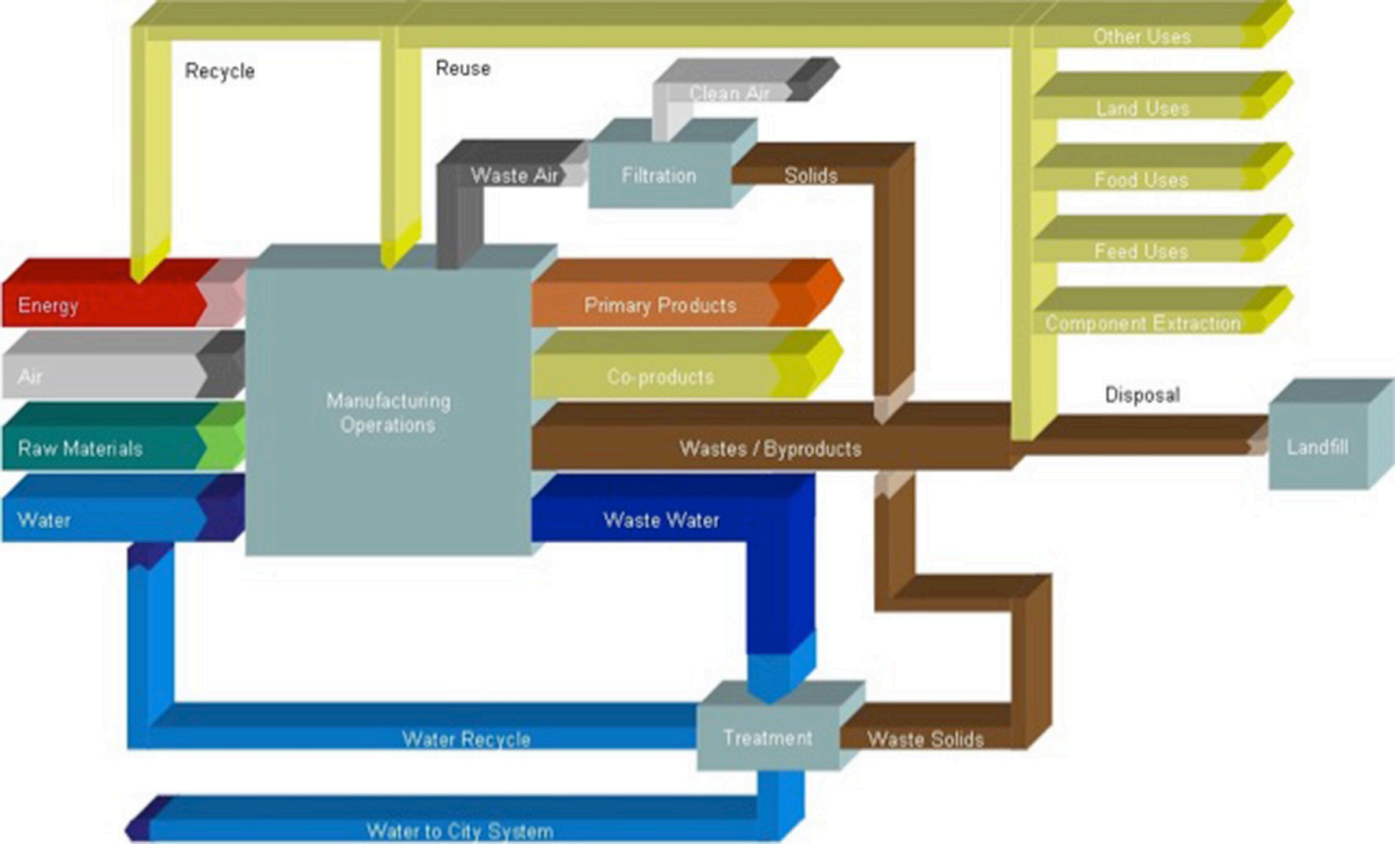
Multiple pathways exist for utilizing waste streams as sources of energy or byproduct applications.

A great quantity of food waste and by-products are generated in many food processing sectors; a few examples include seafood processing (e.g., skin, bones), dairy processing (e.g., whey, curd), vegetable processing (e.g., seeds, skins, shells) and alcohol processing [e.g., brewers' spent grain (BSG), distillers' grains, pomace]. Many of these wastes contain valuable nutrients, such as polysaccharides, vitamins, minerals, fibers, and bioactive compounds such as flavonoids, lycopene, and other carotenoids, which are functional compounds (25) (Figure 5).

Effective utilization of wastes and by-product materials from the meat and poultry industries are of special interest due to high protein and amino acid levels, and high sales prices for these coproducts. High contents of proteins and iron within the blood and other coproducts makes them important edible by-products, which can be used to produce blood sausages, blood pudding, biscuits, and bread in Europe and other regions, as well as blood curd, blood cake, and blood pudding in parts of Asia. Other non-food applications are fertilizer, feedstuff ingredients (e.g., blood meal, meat, and bone meal) and binders. Due to the high foaming capacity of blood plasma, it can be utilized as an alternative for egg whites in baked products (92). Gelatin obtained from animal skins and hides is commonly used in different food and pharmaceutical products, such as meat products (as an emulsifier), ice cream, and other frozen foods (as stabilizers), medicated tablets and pastilles (as binding and compounding agents), and coverings of capsules (25).

Whey is an important by-product of the dairy industry, and contains several valuable constituents, such as proteins (e.g., α-lactalbumin, β-lactoglobulin, and immunoglobulin). In dry form, whey is commonly used in confectionery products, bakery products, and health and sport supplements due to high nutritional (including high amount of essential amino acids) and functional properties (e.g., gelation, foaming, and emulsifying properties) (93). Curd obtained from coagulating milk during curdling processes can be used in probiotic functional foods (25).

Bran is a major by-product of the grain industry, is a significant source of dietary fiber, and is added to various food materials, such as bread, cakes, noodles, pasta, and ice creams. Rice bran has been shown to improve functional and textural properties without altering flavor (94). Wheat bran is commonly used as animal feed. Wheat germ is also a grain byproduct, and can be used in various foods and other products, such as insect biological control agents, pharmaceuticals, and cosmetics (95).

Brewers' spent grain (BSG) is the main by-product of the beer brewing industry and is a source of cellulose and other polysaccharides, in addition to proteins. There are several applications for BSG, such as animal feeds (96), value-added chemicals (e.g., xylitol and lactic acid) (97–99), cultivation of microorganisms such as *Bifidobacterium adolescentis, Lactobacillus* sp. (100), and metal adsorption and immobilization materials for Cu ions (101).

Due to various fruits and vegetables and the broad range of processes, there is a wide range of wastes obtained from fruit and vegetable processing (102). Moreover, depending on plant species, variety, and tissues, several nutritious compounds could be used in different products. Fruit and vegetable by-products offer great potential as a source of additives including antioxidants (e.g., vitamin C), antimicrobials (e.g., phenolic extract), colorants (e.g., anthocyanins), flavorings (e.g., essential oils such as terpenes), and thickening agents (103). Agro-industrial by-products are also reported to be a source of dietary fibers, which are used in the food, cosmetic and pharmaceutical industries (104). Additionally, several fruit by-products such as pineapple waste, grape pomace, and citrus waste could be used to generate ethanol (26), which will be explained in the following section.

#### Energy Generation From Food Waste

In recent years, food waste has become considered to be an untapped resource with much potential for the production of bio-based energy. Energy generation from food waste can be an option to pursue since this approach results not only in lowering the environmental burden of waste disposal, but also in providing energy to the plant, or it can be sold back to the energy grid. The two primary ways for converting food waste to energy include a biological approach (i.e., anaerobic digestion or fermentation) and a thermochemical approach (e.g., gasification, pyrolysis) (26).

Biogas is generated during anaerobic digestion (AD) of organic wastes, and consists mainly of CH_4_ and CO_2_, with trace amounts of other gases [e.g., nitrogen (N_2_), oxygen (O_2_), and hydrogen sulfide (H_2_S)] (105). Murphy et al. (106) demonstrated that 1 m^3^ of biogas from AD is equivalent to ~21 MJ of energy, which can produce ~2 kW h of electricity, assuming 35% conversion efficiency. Key challenges to AD include long process times required for microbial action (~20–40 days), as well as high free ammonia (NH_3_) content (released from the degradation of nitrogen-rich protein compounds), as well as high capital and operations costs (107, 108).

Ethanol production by fermentation of food waste is the other biological approach for converting food waste to energy. The most common microorganism used for this process is *S. cerevisiae*; however, other microorganisms have also been studied, such as *Zymomonas mobilis* and *Pichia rhodanensis* (109, 110). The drawback of *S. cerevisiae* is that it can only use hexose sugars/glucose as a substrate (111), but the other microorganisms can utilize pentose sugars (26). It has also been suggested that waste materials containing high amounts of carbon (e.g., brewery wastes, bran, potato chip waste, etc.) can be good substrates for ethanol production (112).

In terms of conversion of food waste to energy via thermal or thermochemical approaches, there are several methods, such as incineration, pyrolysis, gasification, and hydrothermal oxidation. During incineration, combustion results in heat and energy production from the food waste, which can then be used for operating steam turbines for energy generation, or in heat exchangers for heating up liquid process streams (113, 114). Although incineration can decrease solid waste volume by up to 80–85%, this method is not completely accepted by some countries, and it may even be banned in some countries due to air pollution and toxic air emissions (115).

Pyrolysis is carried out in the absence of oxygen at the temperatures between 250 and 750°C, which then generates bio-oil, syngas (CO + H_2_), and biochar (i.e., residual devolatilized solid waste). Gasification is related to pyrolysis, and it also produces a combustible gas mixture (containing CO, CH_4_, N_2_, H_2_, and CO_2_); but instead of a complete lack of oxygen, the process uses a low content of oxygen, and thus partially oxidizes the food waste at high temperatures (800–900°C). The gas that is produced can be used in engines, and further processing can result in various chemicals (e.g., methanol) (26, 116). Ahmed and Gupta (117) conducted an investigation to compare the performance of pyrolysis to gasification for different properties such as syngas flow rate, output power, and total energy yield; they concluded that gasification was more efficient based on the evaluated criteria, however the gasification process required a longer processing time compared to pyrolysis.

Another thermal conversion technology is hydrothermal carbonization, in which food waste is converted to an energy-rich resource under autogenous pressures, and temperatures ranged from 180 to 350°C. This is a wet process, and offers several advantages, such as lower energy consumption, high waste utilization rates, no odors, relatively short process times (only a few hours), and microorganism destruction due to high processing temperatures (118, 119). Various food waste materials have been examined as substances, including fish meat (120), BSG (121), sweet corn (122), olive pomace (123), peanut shell (124), and grape seed (125).

## Concluding Remarks and Future Directions

The food processing industry consumes large quantities of energy and water. Due to increasing pressure to become more efficient, reduce costs, and reduce environmental impacts, many food processors are implementing technologies to achieve these aims. Energy efficiency has been proven to be greatly improved by replacing current energy- and water-intensive processes with novel, more efficient techniques, such as non-thermal processing. There are many approaches for more efficient water use, including various recycling and reconditioning treatments. Additionally, developing new applications for by-products as well as producing energy from various food wastes can reduce waste and pollution issues. Basic initiatives greatly improve the water efficiency (e.g., install a condensate re-use system, raising staff awareness about proper maintenance and water usage). These trends will continue for the foreseeable future, but their implementation will ultimately be driven by the economics of designing, building, and operating these new unit operations. For additional perspectives, opportunities, and information, the reader is referred to other papers that have been published in this Research Topic.

## Author Contributions

NN and KR searched for and reviewed the literature. NN drafted the paper. KR edited the paper and constructed the figures.

### Conflict of Interest Statement

The authors declare that the research was conducted in the absence of any commercial or financial relationships that could be construed as a potential conflict of interest.
